# Monoclonal Antibodies for Pre- and Postexposure Prophylaxis of COVID-19: Review of the Literature

**DOI:** 10.3390/pathogens11080882

**Published:** 2022-08-05

**Authors:** Serena Vita, Silvia Rosati, Tommaso Ascoli Bartoli, Alessia Beccacece, Alessandra D’Abramo, Andrea Mariano, Laura Scorzolini, Delia Goletti, Emanuele Nicastri

**Affiliations:** Clinical Department, National Institute for Infectious Diseases “Lazzaro Spallanzani” IRCCS, Via Portuense 292, 00149 Rome, Italy

**Keywords:** monoclonal antibodies, COVID-19, prophylaxis

## Abstract

Monoclonal antibodies are laboratory-made proteins that mimic the immune system’s ability to fight off harmful microorganisms, including viruses such as Severe Acute Respiratory Syndrome-CoronaVirus-2 (SARS-CoV-2). The US Food and Drug Administration (FDA) and the European Medical Agency (EMA) have already authorized monoclonal antibodies of anti-SARS-CoV-2 to treat mild to moderate CoronaVIrus Disease-2019 (COVID-19) in patients at risk of developing severe disease. More recently, monoclonal antibodies anti-SARS-CoV-2 have been authorized for primary and secondary prophylaxis in patients at high risk of severe disease for background comorbidity. Primary or pre-exposure prophylaxis prevents COVID-19 in unexposed people, whereas secondary or postexposure prophylaxis prevent COVID-19 in recently exposed people to individuals with laboratory-confirmed SARS-CoV-2. This review focuses briefly on therapeutic indications of currently available monoclonal antibodies for COVID-19 pre- and postexposure prophylaxis and on the efficacy of convalescent plasma.

## 1. Introduction

Antibodies, proteins naturally produced by the body in response to infectious stimuli, are crucial to fight infectious diseases and have been used in the prevention and treatment of bacterial and viral infections for more than a century. The history of medicine counts 5 Nobel prizes for discoveries related to treating infectious diseases with antibodies (Emil Adolf von Behring in 1901), describing humoral immunity (Paul Ehrlich and Il’ja Il’ič Mečnikov in 1908), defining the chemical structure of antibodies (Rodney Robert Porter and Gerald M. Edelman in 1972), the production of monoclonal antibodies (MoAbs) (Niels Kaj Jerne, Georges Köhler, and Cesar Milstein in 1984), and revealing the mechanism of the antibody diversity (Susumu Tonegawa in 1987). In the last 20 years, more than 60 recombinant MoAbs have been developed for human use, including rabies virus, respiratory syncytial virus (RSV), *Clostridioides difficile* (CD), cytomegalovirus, hepatitis B, and are now considered promising for infectious disease targets such as newly emerging viral pathogens (e.g., Ebola, dengue, and Zika) [1,2,3,4]. Their strength lies in their ability to prevent disease progression immediately after administration and to reduce time to recovery, regardless of whether the patient has fully developed immunity. Before the CoronaVIrus Disease-2019 (COVID-19) pandemic, the only licensed MoAbs were palizivumab in 1999 against RSV [5] and bezlotoxumab in 2017 against CD infection [6]. Over the past year, extraordinary scientific, medical, and financial resources have been devoted to the rapid development of diagnostic, prophylactic, and therapeutic measures for Severe Acute Respiratory Syndrome-CoronaVirus-2 (SARS-CoV-2) infection. Anti-SARS-CoV-2 MoAbs are produced in the laboratory and can be derived from B cells of convalescent persons or humanized mice exposed to SARS-CoV-2 antigens; however, MoAbs can be generated by multiple methods, including from the use of samples from vaccinated individuals [7]. The most frequently exploited approach to isolate MoAbs for COVID-19 therapy is to identify B-cell receptors that bind the receptor binding domain (RBD) fragment of the spike (S) glycoprotein with high affinity, since such binding inhibits interactions between RBD and angiotensin-converting enzyme-2 (ACE-2) receptor [8]. RBD domain of protein S binds the ACE-2 receptor, which is present on the surface of cells of the respiratory system, gastrointestinal tract, and endothelium. This binding facilitates the fusion and entry of target cells. Thus, antibodies directed to protein S can neutralize the ability of the virus to bind and fuse with the target host cell [8,9]. Monoclonal antibodies are designed to mimic and precede the body’s natural immune response and are available as prevention and treatment of COVID-19 for patients at high risk of progression to severe disease [10]. They can be used in prophylaxis to prevent disease onset before viral exposure (pre-exposure prophylaxis) and after exposure (postexposure prophylaxis) and, eventually, during infection to prevent disease worsening. The use of MoAbs in the pre-exposure prophylaxis of SARS-CoV-2 infection appears an extremely valid strategy because they act outside the susceptible cell. They reach the maximum efficacy during the early phase of exposure of SARS-CoV-2 with the human host. All antibodies are composed of an antigen-binding fragment (Fab) that provides the specificity to its target and a crystallizable fragment (Fc) that drives the biological function. Changes in both Fab and Fc regions affect the specificity, durability, and the outcome of the antibody-dependent response [4]. Their use in pre-exposure prophylaxis has been made possible by the latest technologies that modify the Fc region of the antibody to extend the MoAb half-life providing potentially protective neutralizing antibody levels for months, depending on the MoAbs required concentrations [11]. 

## 2. COVID-19 Primary Prophylaxis Registered Trials

In June 2021, results from the third part of the BLAZE-2 study, a randomized, double-blind, phase 3 clinical trial designed to evaluate the efficacy of bamlanivimab MoAb in preventing COVID-19 in nursing facility nurses, staff, and residents were published (Table 1). From August to November 2020, 1297 individuals in 74 nursing facilities with at least one confirmed index case of SARS-CoV-2 who had a negative swab at baseline and negative SARS-CoV-2 serology were enrolled. All facility residents fell into the high-risk-for-progression categories, and 41% of staff fell into this category. The study evaluated the results from 966 participants with a mean age of 53 years (74% of them were women), and bamlanivimab was effective in reducing the incidence of SARS-CoV-2 infection compared with placebo (8.5% versus 15.2%) [12]. In August 2021, data supporting the use of AZD7442 MoAbs come from the multicenter randomized (2:1) double-blind phase 3 PROVENT study to evaluate AZD7442 efficacy in pre-exposure prophylaxis. Adults who did not have SARS-CoV-2 infection at baseline and were not vaccinated were enrolled. A total of 5197 participants with a mean age of 53 years (46% of them were women) were recruited, 75% of whom had comorbidities that increased the risk of severe disease. The treatment group consisted of 3460 participants who were given a single 300-mg intramuscular dose of AZD7442 (150 mg of tixagevimab and 150 mg of cilgavimab) and was compared with the placebo group of 1737 participants. The study showed that AZD7442 reduced the risk of developing symptomatic COVID-19 by 77% (95% CI, 46–90) in the treatment group compared with the placebo group. One dose of AZD7442 was shown to provide at least 6 months of sustained protection against SARS-CoV-2 infection [13]. Recently, the novel BA1 omicron Variant of Concern (VoC) of SARS-CoV-2 was sequenced. This VoC presents approximately 30 mutations in the spike protein contributing to increased infectivity and evasion from the immune response produced by vaccines, passive immunization, or natural immunity. In November 2021, casirivimab and imdevimab MoAbs were the first cocktails licensed by EMA for pre-exposure prophylaxis treatment with an indication for adult and adolescent patients with a dosage of 600 mg of casirivimab and 600 mg of imdevimab. It was shown to provide at least 6 months of sustained protection against SARS-CoV-2 infection [14]. In December 21, AstraZeneca reported that the AZD7442 monoclonal antibody combination maintained in vitro neutralizing activity against the omicron VoC [15]. The Emergency Use Authorization (EUA) by the US Food and Drug Administration (FDA) was revised in February 2022 to increase the initial dosing of tixagevimab/cilgavimab for pre-exposure prophylaxis due to immunological escape of tixagevimab/cilgavimab against BA.1 and BA.1.1 Omicron subvariants (while neutralization efficacy against BA.2 subvariant was likely to be preserved) [16]. The amended EUA increased the initial authorized dose to 300 mg of tixagevimab and 300 mg of cilgavimab. In May 2022, the phase 3 (NCT05074433), randomized, double-blind study designed to evaluate the efficacy and safety of casirivimab/imdevimab in preventing COVID-19 in both immunocompromised adolescents and adults is scheduled to close. The study consists of 3 intervention arms with different timing of administration of casirivimab/imdevimab (every four or 12 weeks) compared with placebo with the primary objective of evaluating the cumulative incidence of SARS-CoV-2 infections. Finally, in January 2022, the enrollment for the phase I study COVIDMAB (NCT05135650) started. The main objective of this study is to evaluate the pharmacokinetics of sotrovimab in hematopoietic stem cell transplant recipients. It is planned to administer sotrovimab 1–7 days prior to the start of pre-transplant conditioning. In secondary objectives, the study aims to evaluate the frequency in this population of SARS-CoV-2 infection and therefore its use in this group of patients in primary pre-exposure prophylaxis. Currently, a phase 1, randomized, double-blind, placebo-controlled dose escalation study is underway to evaluate the safety, pharmacokinetics, and immunogenicity of ADM03820 administered as IM injections in healthy adults for the prevention of COVID-19. ADM03820 is a 1:1 mixture of two human IgG1 noncompetitive binding anti-SARS-CoV-2 antibodies [17]. Preliminary data are expected in March 2023. MoAbs tested for the pre of SARS-CoV-2 infection and COVID-19 are listed in Table 1.

## 3. COVID-19 Secondary Prophylaxis Registered Trials 

MoAbs can be used even in postexposure prophylaxis to reduce the chance to get SARS-CoV-2 infection after exposition (Table 1). In June 2021, the trial STORM CHASER, a phase III double-blind, placebo-controlled trial for postexposure prophylaxis of COVID-19 in adults (STORM CHASER) examined the AZD7442 for the ability to prevent symptomatic COVID-19. The safety and efficacy at preventing symptomatic COVID-19 of a 300 mg intramuscular dose of AZD7442 was compared to that of a placebo among 1121 unvaccinated participants with recent (≤8 days) exposure to an individual with laboratory-confirmed COVID-19. The trial was closed considering that it did not meet the primary endpoint of postexposure prevention of symptomatic COVID-19 with AZD7442 compared to placebo [18]. Participants were unvaccinated adults 18 years and over with confirmed exposure to a person with a case of the SARS-CoV-2 virus within the past eight days. In the overall trial population, AZD7442 reduced the risk of developing symptomatic COVID-19 by 33% (95% confidence interval (CI): −26, 65) compared to placebo, a difference not statistically significant. In September 2021, FDA expanded the EUA for bamlanivimab 700 mg and etesevimab 1400 mg administered together to include postexposure prophylaxis in certain individuals for the prevention of SARS-CoV-2 infection. Neutralizing antibodies can now be used together to treat high-risk individuals 12 years of age and older who have not been fully vaccinated against COVID-19 or are not expected to mount an adequate immune response to complete vaccination and have been exposed to someone infected with SARS-CoV-2 or who are at high risk of exposure in an institutional setting, including a nursing home or prison. Data come from the trial BLAZE-2 on bamlanivimab, but in September 2021, because the combination bamlanivimab plus etesevimab has greater antiviral activity than bamlanivimab alone, the FDA presumed it to be effective based on the results of BLAZE-2 [19]. In November 2021, casirivimab/imdevimab MoAbs have been licensed for this use. They can be administered in adults and pediatric individuals (600 mg casirivimab and 600 mg imdevimab doses) who are at high risk for progression to severe COVID-19. These patients, unvaccinated or unexpected to mount an adequate immune response to complete SARS-CoV-2 vaccination, have been exposed by close contact to an individual infected with SARS-CoV-2 or are at high risk of exposure to an individual infected with SARS-CoV-2 because of occurrence of SARS-CoV-2 infection in other individuals in the same institutional setting (for example, nursing homes, prisons) [14]. MoAbs tested for postexposure prophylaxis of SARS-CoV-2 infection and COVID-19 are listed in Table 1.

## 4. Limits to MoAb Use and Alternative Drugs 

Before drawing final conclusions on Moab’s use, several limitations are discussed. There is a wide supply shortage in drug procurement with marked differences even in high income countries, particularly during epidemic peaks of COVID-19. Monoclonal antibodies are almost exclusively available and used in COVID-19 patients from high-income countries only, mainly due to their high cost. The time required between in vitro studies and in vivo registration trials and the regulatory approval up to the routinely clinical use are so prolonged that change trends in circulating VoCs may affect the susceptibility to currently licenced MoAb treatment [20]. The case of casirivimab and imdevimab MoAbs for primary and secondary prophylaxis is paradigmatic: the registration indication was obtained exactly when its use in clinical practice was no longer recommended based on the in vitro susceptibility of circulating VoCs. An interesting alternative is hyperimmune convalescent plasma (CP), which is available in resource-limited countries with no patent restriction and at relatively low cost because many individual donors can provide multiple units. It is obtained from donors recovered from a specific infection. Ideally, it contains polyclonal antibodies directed against the pathogen with sufficient titer and biological activity to provide a passive immunity to the recipient. It provides a diverse mixture of antibodies with different specificities and functions and should be less vulnerable to the emergence of antibody resistance and viral variants. To date, CP has been used in patients with COVID-19 with often unsatisfactory results probably because the clinical trials conducted during the 18-month COVID-19 pandemic are widely heterogeneous in terms of study design and CP administration schedule, selection of donors, and disease and patients characteristics [21]. 

Scientific evidence in favor of the CP use is described in patients treated with high titer plasma transfusion from 3 days to 9 days after the onset of symptoms in outpatient setting [22,23]. To date, there are no indications on the use of CP in pre-exposure prophylaxis but considering the characteristics it could be a valid option especially in periods of limited supply of MoAbs. Currently, the NCT04377672 trial is evaluating this option in immunocompromised children [24]. Additional clinical trials are underway to further examine the CP use as postexposure prophylaxis. Currently, the preprint of a randomized controlled trial on CP used as postexposure prophylaxis against SARS-CoV-2 infection is submitted [25]. Asymptomatic participants aged ≥18 years with close contact exposure to a person with confirmed COVID-19 in the previous 120 h and negative SARS-CoV-2 test within 24 h before transfusion were enrolled. The study demonstrated that CP was safe but did not reduce SARS-CoV-2 infection in participants transfused up to 120 h following exposure [25]. 

## 5. Future Research Topics 

New MoAbs will enter into clinical trials in the next upcoming months, and they will be evaluated for their ability to limit or modify clinical progression of SARS-CoV-2 infection. Establishing MoAb and CP therapeutic and prophylactic efficacy would represent a step forward in controlling the COVID-19 pandemic especially in the most vulnerable groups such as patients with autoimmune, oncohematologic, or neurologic diseases, particularly in resource-limited countries.

## Figures and Tables

**Table 1:** MoAbs tested for the pre- and postexposure prophylaxis of SARS-CoV-2 infection and COVID-19.

	Bamlanivimab	Bamlanivimab/ Etesevimab	Casirivimab/ Imdevimab	Tixagevimab/ Cilgavimab	Sotrovimab	ADM03820
**Pre-exposure prophylaxis**						
**Company**	Eli Lilly (Indianapolis, US)	Eli Lilly (Indianapolis, US)	Roche (Basel, Swiss)	Astra Zeneca (Cambridge, UK)	GSK (London, UK)	Ology Bioservices (Alachua, US)
**Phase**	Phase 3 (completed)	Phase 3 (enrollment not initiated)	Phase 3 (completed)	Phase 3 (completed)	Phase 2	Phase 1
**Efficacy** *(Drug vs Placebo,* *as reported in published RCT*)	Odds Ratio 0.43 (95% CI, 0.28–0.68) Absolute risk difference −6.6 (95% CI, −10.7 to −2.6) [12]	/	Odds Ratio 0.17 (95% 0.09–0.33) Relative risk reduction %: 81.4 [14]	Relative Risk Reduction %: 82.8 (95% CI, 65.8 to 91.4) [13]	/	/
**EMA Approved**	No	No	Yes	Yes	No	No
**FDA Approved**	No	No	No	EUA	No	No
**Population**	≥18 years, nursing home residents and staff with a high risk of SARS-CoV-2 exposure	/	≥12 years, ≥40 Kg high risk of progression, not fully vaccinated or are inadequate immune response and: - high risk of exposure	≥12 years, ≥40 Kg severe allergy to vaccination or immunocompromised	Immunocompromised	Adults ≥18 years
**Posology**	4200 mg	/	600 mg + 600 mg Repeat dose every 4 weeks: 300 mg + 300 mg	FDA 300 mg + 300 mg EMA 150 mg + 150 mg	500 mg	/
**Dosage Adjustment**	/	/	not required	not required	/	/
**Method of administration**	IV infusion	/	SC injection or IV infusion	IM injection	IV infusion	IM injection
**Time after COVID-19 vaccine**	COVID-19 vaccine exclusion criteria	/	NA	2 weeks	28 days	COVID-19 vaccine exclusion criteria
**Post-exposure prophylaxis**						
**Phase**	/	Phase 3 (enrollment not initiated)	Phase 3 (completed)	Phase 3	/	/
**Efficacy** *(Drug vs Placebo,* *as reported in published RCT*)	/	/	Odds Ratio 0.17 (95% 0.09–0.33) Relative risk reduction %: 81.4 [14]	/	/	/
**EMA Approved**	/	No	Yes	No	No	/
**FDA Approved**	/	EUA	EUA	No	No	/
**Population**	/	high risk of progression, not fully vaccinated or are inadequate immune response and: - exposed to SARS-CoV-2 or - high risk of exposure	≥12 years, ≥40 Kg high risk of progression, not fully vaccinated or are inadequate immune response and: - exposed to SARS-CoV-2	Adults with potential exposure to SARS-CoV-2 infection	/	/
**Posology**	/	700 mg + 1400 mg	600 mg + 600 mg	150 mg + 150 mg	/	/
**Dosage Adjustment**	/	Pediatrics <40 Kg (only FDA)	not required	/	/	/
**Method of administration**	/	IV infusion	SC injection or IV infusion	IM injection	/	/

IM intramuscolar, SC subcutaneous, IV intraveneous, EUA Emergency Use Autorizathion.

## Data Availability

Not applicable.
